# Phosphatidylserine liposomes induce a phagosome acidification-dependent and ROS-mediated intracellular killing of *Mycobacterium abscessus* in human macrophages

**DOI:** 10.3389/fcimb.2024.1443719

**Published:** 2024-08-19

**Authors:** Tommaso Olimpieri, Noemi Poerio, Greta Ponsecchi, Gustavo Di Lallo, Marco Maria D’Andrea, Maurizio Fraziano

**Affiliations:** ^1^ Department of Biology, Tor Vergata University of Rome, Rome, Italy; ^2^ PhD Program in Evolutionary Biology and Ecology, Department of Biology, Tor Vergata University of Rome, Rome, Italy

**Keywords:** phosphatidylserine, liposome, cystic fibrosis, *Mycobacterium abscessus*, innate immunity, host-directed therapy

## Abstract

*Mycobacterium abscessus* (Mab) is an opportunistic nontuberculous mycobacterium responsible of difficult-to-treat pulmonary infections in vulnerable patients, such as those suffering from Cystic Fibrosis (CF), where it represents a major cause of morbidity and mortality. Additionally, due to the intrinsic extensive antimicrobial resistance spectrum displayed by this species and the side effects reported for some available antibiotics, the therapeutic management of such infections remains extremely difficult. In the present study, we show that phosphatidylserine liposomes (PS-L) enhance intracellular mycobacterial killing of Mab infected human macrophages with functional or pharmacologically inhibited cystic fibrosis conductance regulator (CFTR), by a mechanism involving phagosome acidification and reactive oxygen species (ROS) production. Additionally, PS-L significantly reduce proinflammatory response of Mab infected macrophages in terms of NF-kB activation and TNF-α production, irrespective of CFTR inhibition. Altogether, these results represent the proof of concept for a possible future development of PS-L as a therapeutic strategy against difficult-to-treat Mab infection.

## 1 Introduction

Cystic Fibrosis (CF) is the most common autosomal recessive genetic disease in the caucasian population (De Boeck, 2020). Mutations in the cystic fibrosis transmembrane conductance regulator (CFTR)-encoding gene lead to the production of defective protein, with severe consequences for almost every organ in the body. The increased secretion of Na^+^ together with the reduced Cl^-^ and water release by CF airways epithelial cells results in a very viscous mucus which limits ciliary clearance and promotes bacterial residence and growth, leading to chronic recurrent lung infections (Boucher, 2002). Additionally, CFTR mutations affect phagocytosis process (Jaganathan et al., 2022), further decreasing bacterial clearance in the airways and opening up the possibility for colonization by opportunistic pathogens, such as *Mycobacterium abscessus* (Mab), which is the most common nontuberculous mycobacterial lung disease etiological agent in CF patients, representing a major cause of morbidity and mortality. Additionally, given the wide intrinsic antimicrobial resistance spectrum of Mab which comprises macrolides, rifamycins, tetracyclines, some β-lactams and aminoglycosides, the therapeutic management of such infections remains extremely long and difficult as there are no optimal drugs, regimens, and therapy duration guidelines currently available (Degiacomi et al., 2019). In this picture, the identification of a novel therapeutic strategy for the management of Mab infections in CF patients, able to replace or integrate antibiotic administration, is highly demanded.

Phagocytosis is the main effector mechanism of antimicrobial innate immune response, and it is mediated by the timely and spacely coordinated expression of second lipid messengers which drive phagosome maturation via the recruitment on the maturing phagosome of effector proteins with specific lipid-binding domains (Saharan and Kamat, 2023). Some pathogens, such as *Mycobacterium tuberculosis*, exploit this mechanism to halt phagosome maturation and thus evading innate immune response (Fratti et al., 2003). On the other hand, the same mechanism can be the target of host-directed therapies aimed at enhancing its protective innate immune response in order to ease the resolution of infections by itself. Considering the well-defined role that lipids have in the phagocytic process, it is possible to modulate phagosome formation and maturation via second lipid messengers’ administration in the form of liposomes which are specifically designed for this purpose (Nisini et al., 2018).

Phosphatidylserine (PS) exposure represents an “eat me” signal normally expressed by apoptotic bodies, which allows their internalization by macrophages in an immunologically silent manner (Li, 2012). However, PS may play additional roles and activate other signaling functions in phagocytosis process that rely on its negative charge (Cauvi et al., 2019). In this context, we have already reported that liposomes made entirely of L-α-phosphatidylserine (PS-L) have an impressive *in vitro* effect on *Mycobacterium tuberculosis*/HIV coinfected macrophages, reducing both *M. tuberculosis* viability and HIV replication, while decreasing pro-inflammatory signals (Poerio et al., 2022a). Considering the state of recurring chronic pulmonary infections of CF patients, and the consequent inflammation-associated tissue damage, which in many cases leads to lung failure (Esther et al., 2010), in this work we sought to determine whether the *in vitro* administration of PS-L to Mab-infected macrophages, whose CFTR has been pharmacologically inhibited or not, might be beneficial in term of enhanced antimicrobial capabilities and re-balanced inflammatory response.

## 2 Method

### 2.1 Cell cultures

The human promonocytic THP-1 leukemia cell line, supplied by the European Collection of Cell Culture, was grown in RPMI 1640 containing fetal bovine serum (10%), gentamicin (5 mg/mL), L-glutamine (2 mM), nonessential amino acids (1 mM), and sodium pyruvate (1 mM) and cultured in 75-cm^2^ polystyrene flasks. Prior to experiments, cells (5x10^5^ cells/mL) were seeded in 24-well plates and induced to differentiate by 72 hrs stimulation with phorbol 12-myristate 13-acetate (PMA) (20 ng/mL), to be used as a model of human macrophages (dTHP-1). Primary monocyte-derived macrophages (MDM) were prepared as previously described (Poerio et al., 2020). Briefly, peripheral blood mononuclear cells (PBMC) from healthy donors were isolated on Ficoll density gradient, and the monocytes were then positively sorted using anti-CD14 monoclonal antibodies conjugated to magnetic microbeads (Miltenyi Biotec) according to manufacturer’s instructions. The monocytes were then suspended in complete medium and incubated for further 5 days in 24-well plates at the concentration of 5x10^5^ cells/mL in presence of macrophage colony-stimulating factor (MCSF) (50 ng/mL) (Miltenyi Biotec) to obtain monocyte-derived macrophages (MDM).

### 2.2 Liposomes

PS-L liposomes were generated via thin layer evaporation technique. Briefly, 35 µg of L-α-phosphatidylserine (Avanti Polar Lipids) were dissolved in trichloromethane and 4 hrs organic solvent evaporation under vacuum at 42°C was carried out via Rotavapor^©^ R-100 (Büchi). Resulting lipid film was hydrated in 1 mL of ultrapure bi-distilled water (Millipore, Merck) by vortex mixing for 10 mins followed by 10 mins of sonication in a sonicating bath. Finally, to achieve a uniform liposome suspension in terms of vesicles dimension, liposomes were extruded 10 times through 0.22 µm polycarbonate membrane (mini-extruder, Avanti Polar Lipids).

### 2.3 Bacteria

*Mycobacterium abscessus* ATCC 19977 (Mab) single colonies were collected by streaking on Middlebrook 7H10 medium (BD Difco) supplemented with oleic acid, albumin, dextrose, and catalase (OADC), then suspended in 15 mL of Middlebrook 7H9 broth (BD Difco) supplemented with albumin, dextrose, and catalase (ADC), Tween80 0,05%, and grown in an Erlenmeyer flask at 37°C under stirring for 40 hrs. Bacterial growth was monitored by measuring optical density at 600nm using a spectrophotometer (Varioskan LUX multimode microplate reader; Thermo Fisher Scientific). For long term storage, bacilli were cryopreserved at -80°C in the Microorganism Preservation System – Protect (Technical Service Consultants Ltd).

### 2.4 Infection and evaluation of intracellular bacterial growth

To assess intracellular bacterial growth, dTHP-1 cells and MDM from healthy donors were distributed in 24-well plates at the concentration of 5x10^5^ cells/mL. Cells were infected with Mab, for 3 hrs at 37°C at a Multiplicity Of Infection (MOI) of 10 in the presence or absence of INH172 (10 mM), a CFTR inhibitor that binds to the nucleotide binding domain-1 (NBD-1) of CFTR leading to a rapid, reversible, and voltage-independent inhibition of the channel (Taddei et al., 2004). Thereafter, extracellular bacilli were killed by a 1 hr incubation with 250 µg/mL amikacin. Cells were then washed and incubated with PS-L (525 ng) for further 18 hrs in presence or absence of INH172 (10 mM). Finally, cells were lysed with 1% deoxycholate (Sigma), samples were diluted in PBS-Tween 80 0.05%, and Colony Forming Units (CFU) quantified by plating bacilli in triplicate on 7H10.

To evaluate the role of Reactive Oxygen Species (ROS) and of phagosome acidification in intracellular bacterial killing, MDM were infected as described above and then treated or not for 18 hrs with PS-L (525 ng) in conjunction with either polyethylene glycol (PEG)-catalase (PEG-Cat) at 100 U/mL or concanamycin A (Conc A) at 1 nM. Finally, cells were lysed with 1% deoxycholate (Sigma), samples were diluted in PBS-Tween 80 0.05%, and CFU quantified by plating bacilli in triplicate on 7H10.

### 2.5 Fluorimetric analysis

For the evaluation of phagosomal acidification during infection, Mab was stained with 5(6)-Carboxyfluorescein N-hydroxysuccinimide ester (NHS; Sigma), a pH-sensitive probe able to bind to Mab envelope and whose fluorescence is directly proportional to pH. Briefly, 7H9 grown Mab was pelleted at 14000x*g* for 10 mins and stained in PBS Tween 20 0.05% containing NHS 100 µg/mL for 30’ at +4°C. Subsequently Mab was washed twice via centrifugation in PBS Tween 20 and finally used to infect MDM from healthy donors (INH172-inhibited or not) at MOI 10 for 3 hrs at 37°C, 5% CO_2_. After infection, MDM were washed twice with RPMI without phenol red to remove all extracellular bacteria and to dilute any traces of phenol red which may interfere with readings and finally cells were stimulated with PS-L (525 ng) for 18 hrs. A pH calibration curve was obtained by incubating MDMs in calibration buffers at fixed pH values of 4.4, 5.5, 6.5, and 7.5 (**Supplementary Figure 1**) as per manufacturer instructions (intracellular pH calibration buffer kit; Molecular Probes). To reduce background noise generated from FBS present in complete RPMI, before readings the medium in wells was replaced with PBS and then fluorescence intensity determined at λ_ex_ 492 nm and λ_em_ 517 nm using a Varioskan LUX multimode microplate reader (Thermo Fisher Scientific).

For ROS generation assessment, MDMs ± INH172 were infected with Mab as described in “Infection and evaluation of intracellular bacterial growth” and then loaded with the fluorescent indicator 20,70-Dichloro-dihydro-fluorescein diacetate (DCFH-DA) (Molecular Probes) 10 µM via incubation for 40 mins in the dark at 37°C. Subsequently cells were treated with PS-L (525 ng) and fluorescence determined after 4 hrs at λ_ex_ 488 nm and λ_em_ 530 nm using a Varioskan LUX multimode microplate reader (Thermo Fisher Scientific).

### 2.6 Enzyme-Linked Immunosorbent Assay

NF-κB activation was determined by NFκB p65 (Total) Human InstantOne™ ELISA Kit (Invitrogen). Briefly, dTHP-1 cells or MDMs ± INH172 were infected with Mab as described in “Evaluation of bacterial intracellular growth”. Cells were lysed as per manufacturer instructions right after infection or after 1 hr PS-L (525 ng) treatment and lysates were stored at -20°C until analysis. NF-κB activation of stored samples was evaluated according to manufacturer instructions using Varioskan LUX multimode microplate reader (Thermo Fisher Scientific). For tumor necrosis factor-α (TNF-α) levels quantification, MDMs ± INH172 were infected or not with Mab as described in “Evaluation of bacterial intracellular growth” After infection extracellular bacilli were removed by 1 hr incubation with Amikacin 250 µg/ml and finally stimulated or not with PS-L (525ng) for 3 hrs. Thereafter, supernatants were collected, all possible remaining bacteria or cellular debris removed by 5 mins centrifugation at 14000x*g*, and finally samples were stored at -20°C until analysis. The levels of TNF-α were measured by human TNF-α DuoSet^®^ ELISA Development Systems (R&D Systems, Minneapolis, MN, USA) as per manufacturer’s instructions.

### 2.7 Ethics statement

Buffy coats from anonymized healthy donors, who gave their written informed consent to donate the non-clinically usable components of their blood for scientific research, were obtained from the Blood Transfusion Unit of “Policlinico Tor Vergata” in Rome, Italy (ethics approval no. 16/2020).

## 3 Results

### 3.1 PS-L enhances intracellular killing of *M. abscessus* in dTHP-1 cells and in INH172-inhibited or not monocyte-derived macrophages (MDM)

**Figure 1:**
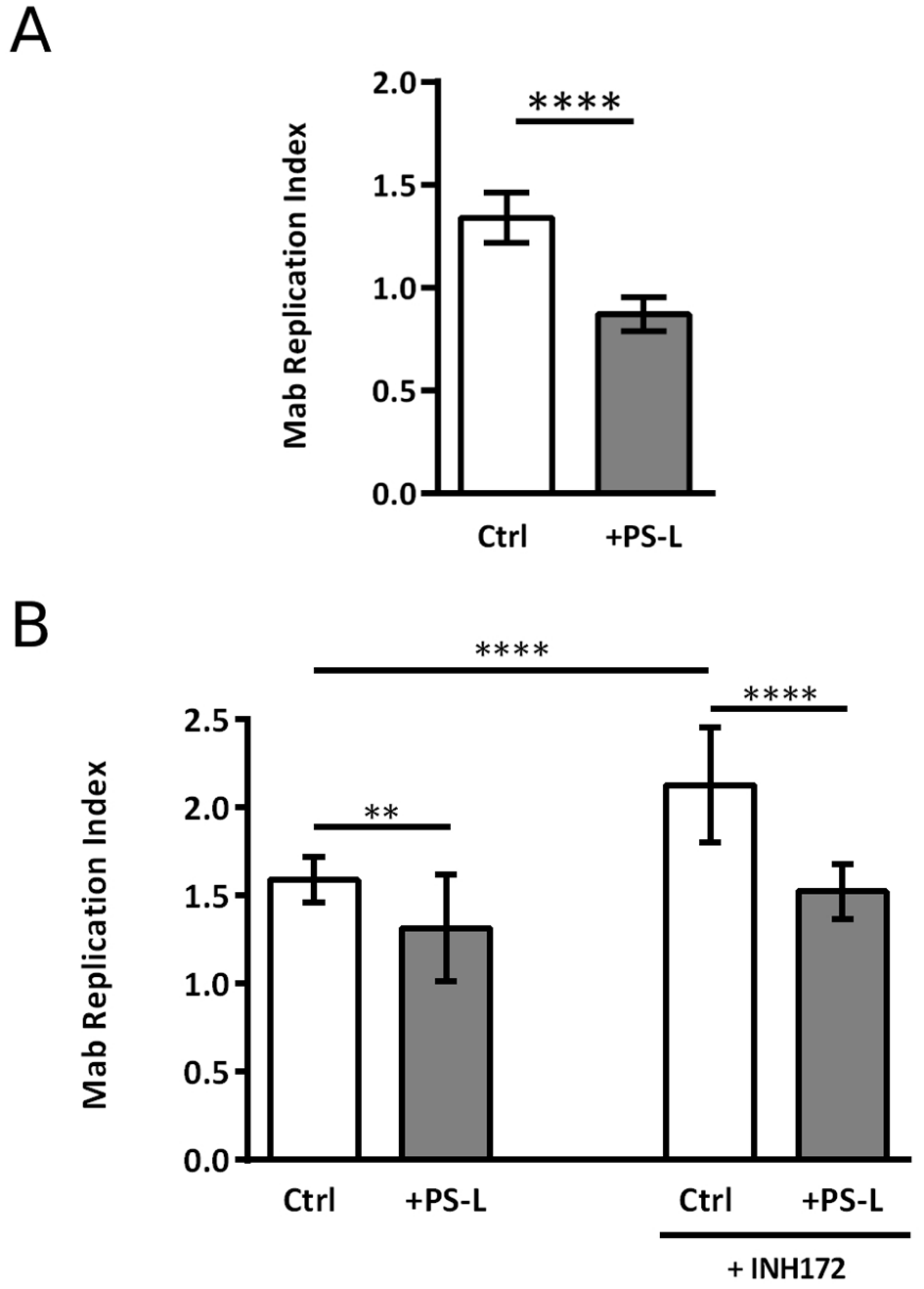
PS-L treatment enhances antimycobacterial response in *M. abscessus* infected dTHP-1 cells and MDM treated or not with INH172. **(A)** dTHP-1 cells or **(B)** monocyte derived macrophages, treated or not with INH172 were infected or not with Mab (MOI 10) for 3 hrs at 37°C, then extracellular bacilli were killed by 1 hr of incubation with amikacin 250 µg/ml. Finally, cells were treated for another 18 hrs with PS-L. Bacterial growth was assessed by CFU assay. Replication index was calculated as the ratio between the CFU obtained after 18 hrs from infection in the presence or absence of PS-L and the CFU obtained at time 0, before the addition of PS-L. The results are shown as mean ± standard deviation of the values obtained from triplicate of each condition and are representative of three independent experiments. ** p<0.01; **** p< 0.0001 by two tailed Student’s test.

Given the promising effects that phosphatidylserine liposomes (PS-L) administration proved to have on *M. tuberculosis*/HIV coinfected macrophages (Poerio et al., 2022a), the same liposome formulation was chosen to test its *in vitro* capability of enhancing the intracellular killing of Mab. In this context, preliminary experiments of dose dependency were performed to identify the optimal amount of PS-L to be used for *in vitro* evaluation of pro-microbicidal activity. Results, expressed in **Supplementary Figure 2**, show 525 ng of PS-L as the optimal amounts of liposomes. Thereafter, the *in vitro* therapeutic value of PS-L formulation was tested in a more comprehensive study by using dTHP-1 cells as well as primary macrophages infected with Mab. Results, expressed in **Figure 1A**, confirm a significant increase of intracellular mycobacterial killing after PS-L stimulation in Mab-infected dTHP-1 cells when compared to Mab infected control cells. We further explored the capability of PS-L to restrict the intracellular mycobacterial growth in Mab infected primary macrophages and in primary macrophages whose CFTR has been pharmacologically inhibited with INH172. Results show that PS-L stimulation of Mab infected primary macrophages results in a significant reduction of intracellular mycobacterial viability, irrespective of CFTR inhibition (**Figure 1B**).

### 3.2 PS-L enhances intracellular *M. abscessus* killing via induction of ROS production and phagosome acidification in MDM treated or not with INH172

The antimycobacterial response induced by PS-L in dTHP-1 cells and primary macrophages was deeper investigated in terms of mechanisms responsible of intracellular mycobacterial killing. After bacterial internalization, a rather complex multi-step process takes place to promote maturation of early immature phagosomes for their maturation into highly microbicidal phagolysosomes. During this process, phagosomes progressively acidify and assembles type II NADPH oxidase (NOX-2) subunits in order to form fully active enzyme responsible of ROS generation in mature phagolysosomes. On these grounds, we investigated if the observed PS-L induced antimicrobial response was due to a mechanism involving phagosome acidification and ROS production (Lee et al., 2020). First, to effectively track changes in phagosomal pH after Mab engulfment, bacteria were stained with NHS-carboxyfluorescein and used to infect MDM treated or not with INH172. NHS is a pH sensitive fluorescent probe revealing pH value after comparing fluorescence values with those obtained from a pH calibration curve.

**Figure 2:**
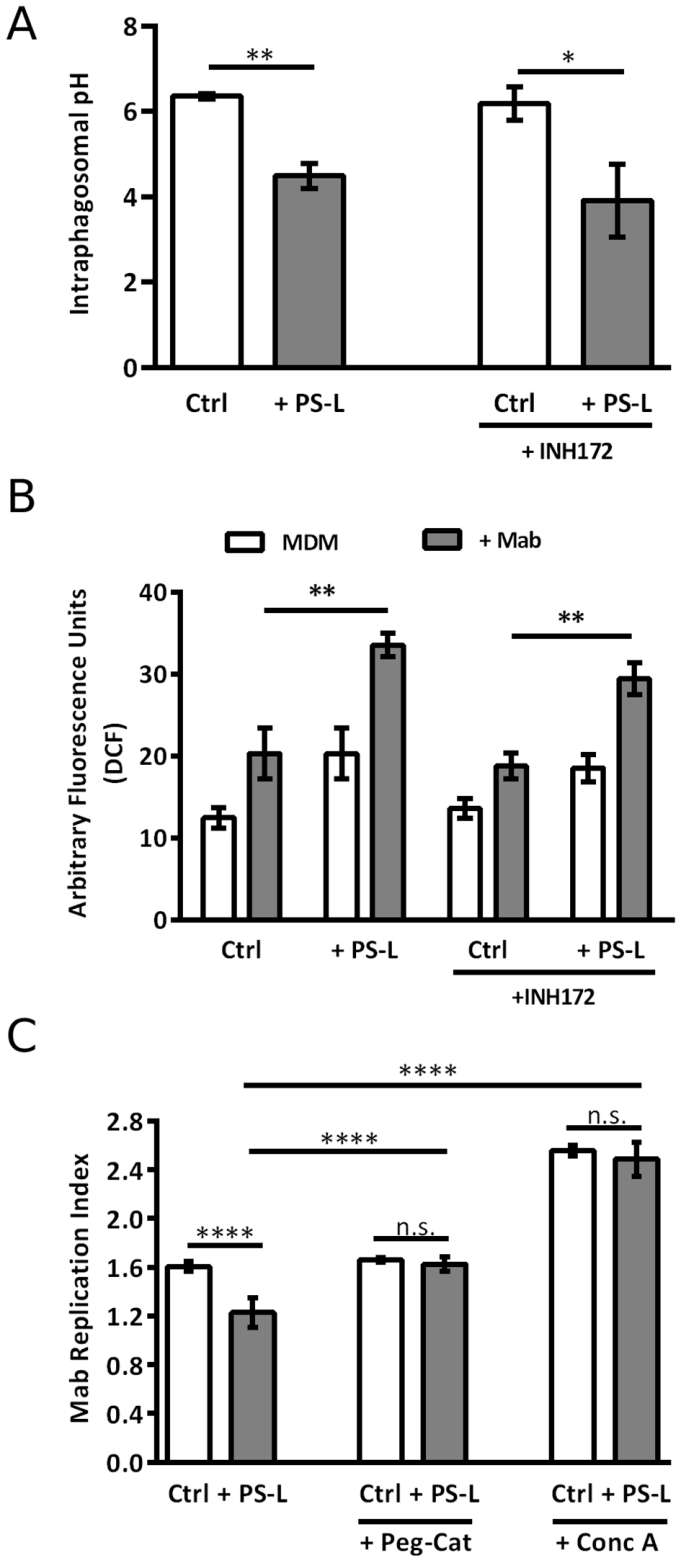
PS-L treatment enhances MDM antimycobacterial response by increasing phagosomal acidification and ROS production. **(A)** Mab was labeled with 100 mg/ml of the pH sensitive dye NHS and used (MOI 5) to infect MDM for 3 hrs at 37°C, then extracellular bacilli were killed by 1 hr incubation with amikacin 250 μg/ml. Infected cells were then treated with PS-L for 18 hrs. **(B)** MDM were infected with Mab (MOI 10) for 3 hrs at 37°C, then extracellular bacilli were killed by 1 hr incubation with amikacin 250 μg/ml. After, cells were labeled with DCF 10mM and subsequently treated with PS-L for 4 hrs. The intensity of DCF fluorescence is proportional to the amount of ROS produced. **(C)** MDM were infected with Mab (MOI 10) for 3 hrs at 37°C, then extracellular bacilli were killed by 1 hr incubation with amikacin 250 μg/ml. Finally, cells were treated for another 18 hrs with PS-L in combination with Conc A (1 nM) or PEG-Cat (100 U/mL). Bacterial growth was assessed by CFU assay. Replication index was calculated as the ratio between the CFU obtained after 18 hrs from infection and the CFU obtained at time 0, before the addition of PS-L. Results are shown as mean ± standard deviation of the values obtained from triplicate cultures and are representative of three independent experiments. *p<0.05; ** p< 0.01; **** p< 0.0001 by two tailed Student’s t test.

Results show that, at 18 hrs post-infection, PS-L induced a more acid phagosomal lumen when compared to untreated controls, in Mab infected MDM, irrespective of CFTR inhibition (**Figure 2A)**. Phagosome acidification and ROS generation are sequential steps leading to intracellular bacterial killing. As NOX-2 assembles from component subunits on maturing phagosomes (Nunes et al., 2013), we monitored ROS generation following stimulation with PS-L in MDM with constitutive or pharmacologically inhibited CFTR. In this context, in accordance with the enhancement of phagosome maturation, PS-L stimulation induced significantly higher amounts of ROS in Mab infected MDM, irrespective of CFTR pharmacological inhibition, as detected by the cell permeable redox probe DCFH-DA (**Figure 2B**).

To further validate these results, Mab-infected MDM were exposed to Conc A, an inhibitor of V-ATPase (Huss et al., 2002), or to PEG-Cat, which converts hydrogen peroxide to water and oxygen, thus reducing ROS activity. Results indicate that both Conc A and PEG-Cat (**Figure 2C**) completely abolish the effect that PS-L on Mab replication index, demonstrating that the main mechanism of Mab intracellular killing enhancement of such liposome formulation relies on phagosome acidification and ROS generation.

### 3.3 PS-L reduces NF-κB activation and TNF-α production in dTHP-1 cells and INH172-inhibited or not MDM infected with *M. abscessus*

**Figure 3:**
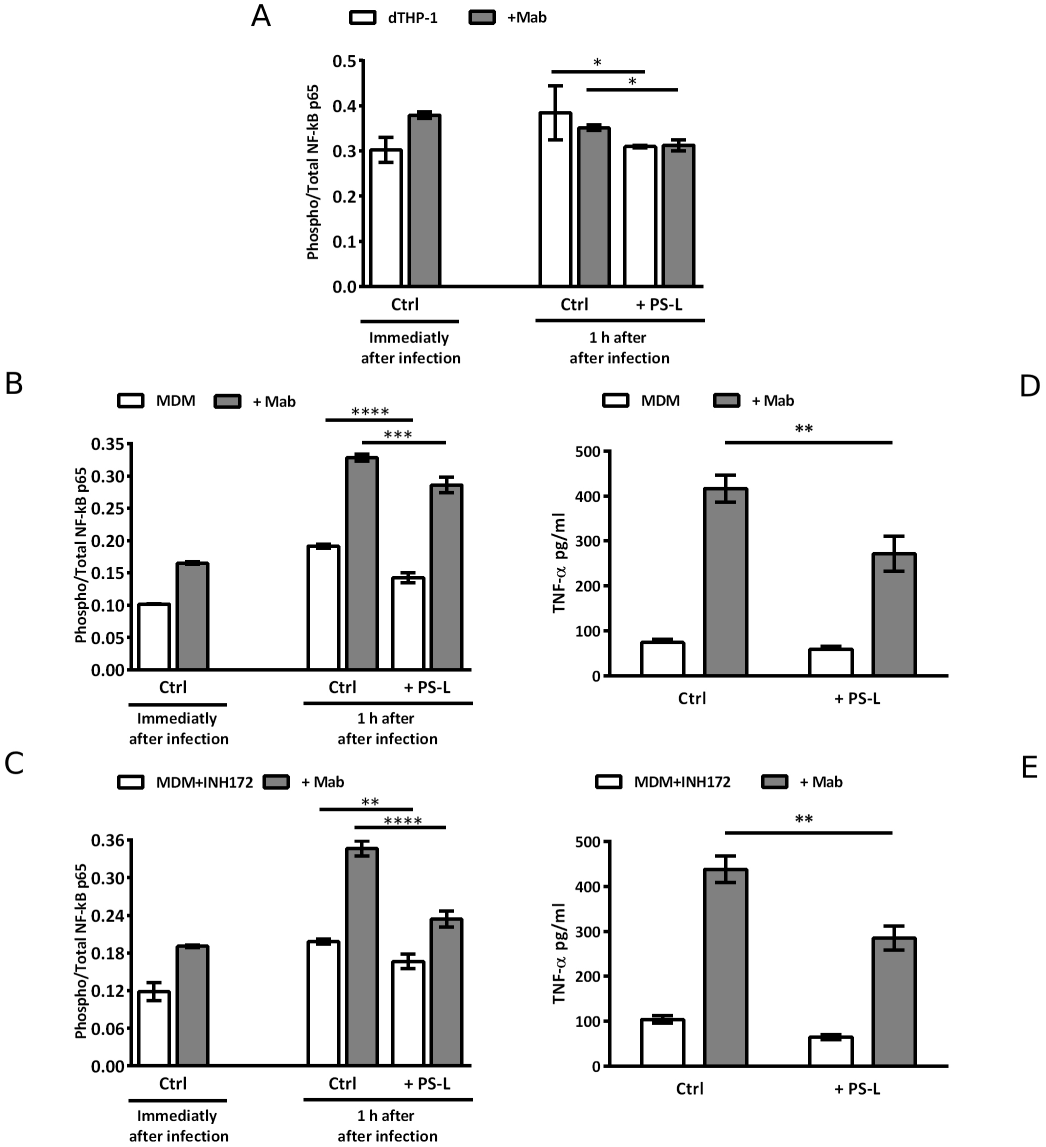
PS-L induces significant reduction of NF-kB activation and TNF-α production in dTHP-1 and in MDM *M. abscessus*-infected treated or not with INH172. dTHP-1 cells **(A)**, MDM **(B, D)**, or MDM+INH172 **(C, E)** cells were infected or not with Mab (MOI 10) for 3 hrs at 37°C, then extracellular bacilli were killed by 1 hr incubation with amikacin 250 µg/ml. **(A–C)** Cells were then treated or not with PS-L for 1 hr and then lysed and stored. NF-κB activation is expressed as the ratio Phosphorylated/Total NF-κB. For baseline NF-kB levels determination, cells were lysed and stored immediately after antibiotic treatment. **(D, E)** Cells were treated or not with PS-L for 3 hrs and then supernatants were harvested and stored until analysis. The production of TNF-α was analyzed by ELISA. Results are shown as mean ± standard deviation of the values obtained from triplicate cultures and are representative of three independent experiments. *p<0.05; ** p< 0.01; *** p< 0.001; ****p<0.0001 by two tailed Student’s t test.

Chronic and unresolved acute Mab infections in CF patients can cause progressive inflammatory lung damage (Esther et al., 2010; Floto et al., 2016). Given the widely known anti-inflammatory function of PS (Nagata, 2018), and considering the previous results regarding anti-inflammatory effects of liposomes containing PS (Greco et al., 2012; Poerio et al., 2020, 2022b), we investigated the effects of PS-L stimulation on NF-κB activation in dTHP-1 cells and in MDM exposed or not to CFTR inhibitor INH172, and infected or not with Mab. As expected, Mab infection caused a spike in NF-κB activation in macrophage cell line (**Figure 3A**) and in primary MDM (**Figures 3B, C**). However, PS-L treatment induced a significant reduction in NF-κB activation, with Mab infected primary macrophages undergoing pharmacological CFTR inhibition displaying the most significant effect (**Figure 3C**). The reduced activation of NF-κB was then confirmed by the analysis of TNF-α production, whose transcription specifically depends upon NF-κB activity (Deree et al., 2008), as other pro-inflammatory cytokines production such as IL-1β or IL-6 may be induced by alternative pathways (Tanaka et al., 2014; Pyrillou et al., 2020). As expected, Mab infected MDM showed a significant increase in TNF-α at 3 hrs after infection in comparison with uninfected macrophages, and PS-L treatment reduced the levels of the cytokine both in uninfected and infected cells, irrespective of CFTR inhibition (**Figures 3D, E**).

## 4 Discussion

*M. abscessus* (Mab) infections still remain a worrisome event in clinical practice mainly due to the intrinsic antibiotic resistance of the pathogen and to its ability to acquire novel resistances. In fact, Mab is equipped with a waxy impermeable cell wall, drug export systems, and drug- or drug target- modifying enzymes which make it resistant to a wide range of antimicrobial classes (Nguyen et al., 2024). Additionally, current standard pulmonary antibiotic therapy for Mab infections, especially in CF patients, relies on long-term, multiphasic administration of antibiotics which is divided in an intensive phase (3–12 weeks), consisting in daily oral macrolide treatment together with intravenous administration of amikacin and one among tigecycline/imipenem/cefoxitin, followed by a continuation phase (at least 1 year) which includes inhaled amikacin in conjunction with 2–3 antibiotics oral daily administration (Johansen et al., 2020). Such a prolonged and intensive antibiotic treatment presents some major problems including i) efficacy dependent on patient compliance with drug regimen, ii) frequent events of drug-intolerance or drug-related toxicity (Sánchez-Borges et al., 2013), and iii) Mab acquired antimicrobial resistance due to spontaneous mutations. Thus, the discovery of additional and more effective anti-Mab drugs and/or the identification of novel therapeutic strategies are urgently needed for the clinical management of these infections.

CFTR mutations in CF patients strongly impair macrophage’s ability to perform an effective bacterial clearance, affecting all stages of phagocytosis. Loss of CFTR leads to a blunted PI3K/AKT signaling which plays an important role in phagocytosis initiation and inflammatory response regulation (Oviedo-Boyso et al., 2011; Di Pietro et al., 2017). Additionally, CF macrophages show evidence of an impaired phagolysosome maturation due to CFTR-related block of phagosomal acidification (Di et al., 2006).

Apart from CF-related phagocytosis impairment, Mab itself has been demonstrated to actively inhibit phagosome maturation and to promote phagosome escape strategies. The *M. abscessus* colonization of lung alveoli begins with smooth (S) strains producing glycopeptidolipids and biofilm, while in the invasive infection, rough (R) mutants are more virulent and responsible of severe disease. Moreover, Mab S phenotype is more prone to phagocytosis and, more importantly, is capable both to inhibit phagosome acidification and maturation (Roux et al., 2016) and to escape to the cytosol by causing phagosome membrane rupture, via the ESX-4 locus (Laencina et al., 2018). Given the features of S morphotype and considering that the focus of the present manuscript was to restore and/or enhance the phagosome maturation process, actively inhibited by the pathogen or impaired due to CFTR mutations, we prioritized the study on Mab S phenotype. In this context, the development of a host-directed therapy aimed at enhancing the phagosome maturation process could represent a valuable therapeutic strategy to counteract host-impaired and pathogen-inhibited phagosome maturation and hence to restore an efficient mycobactericidal activity in CF macrophages.

We already identified and validated several second lipid messengers involved in the phagosome maturation process which can enhance innate (myco)bactericidal response when delivered to macrophages by apoptotic body-like liposomes (ABLs) (Poerio et al., 2017, 2020). Interestingly, ABLs loaded with phosphatidylinositol-5-phosphate can enhance Mab killing both in impaired macrophages from CF patients, and in an *in vivo* murine model of chronic infection (Poerio et al., 2022b). Recently, we reported that liposomes made entirely of phosphatidylserine (PS-L) succeeded in reducing intracellular mycobacterial viability and HIV replication in *Mycobacterium tuberculosis* (MTB)/HIV coinfected macrophages (Poerio et al., 2022a). In the present study, we wanted to assess the *in vitro* therapeutic value of PS-L on Mab infected human MDM, with or without pharmacological inhibition of CFTR. Our results clearly indicate that administration of PS-L to Mab infected macrophages significantly enhances their antimicrobial activity leading to intracellular mycobacterial killing, irrespective of CFTR functionality, further supporting and extending the therapeutic value of PS-L, previously reported in the context of MTB/HIV infection, also in the context of the infection with the fast growing, difficult-to-treat, Mab.

It has been already reported that PS, as an anionic phospholipid, alters phagosome membrane charge and interacts with polycationic motifs of several effector proteins, activating a downstream signaling which triggers innate immune response (Cauvi et al., 2019). This binding ability is an important event which regulates the distribution of both synaptotagmins and small GTPases of the Rab and Rho superfamilies, with the former dictating membrane fusion events (Honigmann et al., 2013) and the latter regulating both phagosome formation and maturation (Nisini et al., 2018).

Our findings confirm the capability of Mab to arrest phagosome maturation, blocking phagosome pH near neutrality. However, PS-L stimulation promotes phagosome acidification and ROS production, indicating a recovery of the phagosome maturation process. Moreover, the involvement of these processes in PS-L-induced intracellular Mab killing was confirmed by the evidence that in the presence of Conc A or PEG-Cat the effect of PS-L on intracellular mycobacterial viability was completely abrogated. Furthermore, the evidence that PS-L was capable of restoring the correct phagosome acidification and promoting ROS production also in CF-like macrophages, support the possibility of a future development of PS-L as a host-directed therapeutic strategy for the treatment of Mab also in CF patients.

The role of PS as an anti-inflammatory eat-me signal in phagocytes has been described (Nagata, 2018). Recognition of exposed PS molecules, via the bridge glycoproteins protein S and growth-arrest-specific 6, activates the TAM receptor tyrosine kinases present of the surface of many phagocytes, causing a downstream signaling which inhibits pro-inflammatory cytokine production during efferocytosis (Rothlin et al., 2007). The results reported herein further support and extend the anti-inflammatory properties of PS on Mab-infected CF-like MDM, already reported in MTB/HIV coinfected macrophages.

Lastly, it is worth noting that liposomes, due to their nature of enclosed phospholipids bilayer micelles, have a high biocompatibility, biodegradability, low toxicity, and great ability to encapsulate hydrophilic and hydrophobic compounds, thus representing one of the most successful drug carrier systems known to date (Nisini et al., 2018). Many liposome formulations for the treatment of cancer, fungal or viral infections, and pain management are available for clinical use, and many other formulations are being evaluated (Bulbake et al., 2017). In this context, the possibility of encapsulating antimicrobial compounds within our liposome formulation may present the unique advantage of bringing together the immunomodulatory function presented in this work with the specific antimicrobial action of the drug, in a single formulation. For instance, the intracellular localization of bacterial pathogens may drastically lower antibiotic efficacy by reducing the local drug active concentration due to poor antibiotic permeability, or by colonizing intracellular compartments that are difficult for the drug to reach (McOrist, 2000). Thus, PS-L may serve as drug carrier allowing to achieve a higher intracellular antimicrobial concentration by simultaneously exerting its intrinsic function of phagocytosis enhancer. The synergy resulting from the host- and pathogen- directed approach resulting from drug-loaded PS-L administration may translate to a reduction of the time of therapy and side effects often associated to antimicrobial therapeutic regimen adopted for Mab infection.

## Data Availability

The raw data supporting the conclusions of this article will be made available by the authors, without undue reservation.
