# Supplementary material for: Phosphatidylserine liposomes induce a phagosome acidification-dependent and ROS-mediated intracellular killing of Mycobacterium abscessus in human macrophages
Source: Front Cell Infect Microbiol. 2024 Aug 19;14:1443719. doi: 10.3389/fcimb.2024.1443719 (PMC11366698; doi:10.3389/fcimb.2024.1443719)
Supplement: Supplementary file 1 [file SupplementaryFile1.docx]

Supplementary Material

Phosphatidylserine liposomes induce a phagosome acidification-dependent and ROS-mediated intracellular killing of *Mycobacterium abscessus* in human macrophages

**Olimpieri T.^1^, Poerio N.^1^, Ponsecchi G.^1,2^, Di Lallo G.^1^, D’Andrea M.M.^1^, Fraziano M.^1*^**

*** Correspondence:** Maurizio Fraziano fraziano@bio.uniroma2.it

# Supplementary Data

Method

RFU/pH calibration curve

For the generation of a Relative Fluorescence Units (RFU)/pH calibration curve, Mab was firstly stained with 5(6) Carboxyfluorescein N-hydroxysuccinimide ester (NHS; Sigma). Briefly, 7H9 grown Mab was pelleted at 14000x*g* for 10 mins and stained in PBS Tween 20 0.05% containing NHS 100 µg/mL for 30’ at +4 °C. Subsequently Mab was washed twice via centrifugation in PBS Tween 20 and finally used to infect MDM from healthy donors (INH172-inhibited or not) at MOI 10 for 3 hrs at 37° C, 5% CO_2_. After infection, MDM were washed twice with RPMI without phenol red to remove all extracellular bacteria and to dilute any traces of phenol red which may interfere with readings. A pH calibration curve was obtained by incubating MDMs in calibration buffers at fixed pH values of 4.4, 5.5, 6.5, and 7.5 as per manufacturer instructions (intracellular pH calibration buffer kit; Molecular Probes). To reduce background noise generated from FBS present in complete RPMI, before readings the medium in wells was replaced with PBS and then fluorescence intensity determined at λ_ex_ 492 nm and λ_em_ 517 nm using a Varioskan LUX multimode microplate reader (Thermo Fisher Scientific). Background noise was further reduced by subtracting the signal generated by MDMs (INH172 inhibited or not) infected with non-stained Mab. Finally, a linear regression line was calculated, and its equation was used to convert RFUs from experimental conditions in pH values.

PS-L Dose-dependency assay

To determine the optimal dosage of PS-L to be used as treatment, dTHP-1 cells were distributed in 24-well plates at the concentration of 5x10^5^ cells/mL and infected with Mab, for 3 hrs at 37 °C at a Multiplicity Of Infection (MOI) of 10. Thereafter, extracellular bacilli were killed by a 1 hr incubation with 250 µg/mL amikacin. Cells were then washed and incubated with PS-L 262,5ng, 525ng, 1050ng, and 2100ng for further 18 hrs. Finally, cells were lysed with 1% deoxycholate (Sigma), samples were diluted in PBS-Tween 80 0.05%, and Colony Forming Units (CFU) quantified by plating bacilli in triplicate on 7H10.

# Supplementary Figures

**Supplementary Figure 1.** **RFU/pH calibration curve.**
MDMs (INH-172 inhibited or not) were infected with NHS-stained *M. abscessus* (MOI 10) for 3 hrs at 37 °C, then extracellular bacilli were killed by 1 hr incubation with amikacin 250 µg/ml. Finally, cells were incubated with pH calibration buffers and signal intensity determined at λ_ex_ 492 nm and λ_em_ 517 nm. Thereafter the linear regression for either MDM or MDM+INH-172 was calculated. The results are shown as mean ± standard deviation of the values obtained from triplicate of each condition and are representative of two different experiments.

**Supplementary Figure 2. PS-L dose-dependency assay.**dTHP-1 cells were infected with Mab, for 3 hrs at 37 °C at a Multiplicity Of Infection (MOI) of 10. Thereafter, extracellular bacilli were killed with 1 hr incubation with 250 µg/mL amikacin. Cells were then washed and incubated with PS-L 262,5ng, 525ng, 1050ng, and 2100ng for further 18 hrs. Bacterial growth was assessed by CFU assay. Replication index was calculated as the ratio between the CFU obtained after 18 hrs from infection and the CFU obtained at time 0, before the addition of PS-L. Results are shown as mean ± standard deviation of the values obtained from triplicate cultures.
ns p> 0.05; *** p< 0.001 by two tailed Student’s t test.
